# Part-time work and other occupational risk factors for suicide among working women in the Swiss National Cohort

**DOI:** 10.1007/s00420-020-01629-z

**Published:** 2021-02-01

**Authors:** Pascal Wild, Nicolas Bovio, Irina Guseva Canu, for the Swiss National Cohort, Matthias Egger, Adrian Spörri, Marcel Zwahlen, Milo Puhan, Matthias Bopp, Martin Röösli, Michel Oris, Murielle Bochud

**Affiliations:** 1 Institut National de Recherche et Sécurité (INRS), Vandoeuvre lès Nancy, France; 2 Department of Occupational and Environmental Health, Center for Primary Care and Public Health (Unisanté), Lausanne, Switzerland

**Keywords:** Suicide mortality, Epidemiology, Cohort study, Female, Occupational factors, Part-time work

## Abstract

**Objective:**

The aim of this study was to describe the factors associated with mortality by suicide among working women focusing on work-related factors.

**Methods:**

The study population consisted in all Swiss residents recorded in the 1990 and/or the 2000 compulsory national censuses and were linked to emigration and mortality registers. We selected all women aged 18–65 and at work at the official census dates. Following work-related variables were available: socio-economic status, weekly hours of work, the sector of activity and the job title coded according to the International Standard Classification of Occupations (ISCO). The risk of suicide was modelled using negative binomial regression.

**Results:**

The cohort comprised 1,771,940 women and 2526 deaths by suicide corresponding to 24.9 million person-years. The most significant non-occupational predictors of suicide were age, period, civil status, religion, nationality and geographical regions. Adjusted on these factors, part-time work was associated with increased suicide rates. According to job codes, health and social activities, in particular care-worker had the highest suicide risks.

**Conclusion:**

Suicide among working women depended on work-related factors even taking into account other socio-demographic factors.

## Introduction

For a long time, suicide was considered a “male problem”. However, recent work has highlighted the importance of the phenomenon in both sexes. Suicide being a multifactorial event; different determinants have been identified (FOPH 2005). In both men and women, in all age groups, psychiatric morbidity is the most frequent risk factor, followed by psychosocial factors. Women-specific suicide risk factors have been rarely examined, as women are underrepresented in post-mortem studies compared to men. The few studies indicate women-specific risk factors related to reproduction, including traumatic events such as miscarriage, infant death at birth, or premenstrual syndrome. In addition, there are a range of psychosocial factors to which women are more exposed to, such as among others sexual violence and domestic violence (Beautrais 2006). Among the social determinants of suicides in women of working age (18–64), relationship and family problems, divorce or separation, high levels of urbanisation, and certain aspects of work activity have been identified as risk factors. With regard to the latter, there is very few specific data as studies on the socio-professional determinants of suicide focus almost exclusively on the male population. Given that these determinants are subject to temporal and social changes, and that the number of professionally active women is increasing, it is important to study their role in women's suicides. In our descriptive study of the Swiss population of working age, stratified by sex (Guseva Canu et al. 2019), we observed distinct patterns of suicide mortality between the two genders and identified occupational groups at high and low risk of suicide among men and women.

The aim of the present work is to investigate the female-specific occupational determinants of suicides, by conducting in-depth modelling analyses, taking into account the individual, social, and organizational characteristics of the Swiss female population at work.

It thus completes the comparison of the mortality by suicide to the general population according to job titles (Guseva Canu et al. 2019) by taking into account non-occupational risk factors as well as characteristics of the job.

## Methods

### Population

The target population of this study are all female workers aged over 18 since 1990 living in Switzerland. In practice, the study population is based on the subjects identified in the Swiss National Cohort described (SNC). The SNC is described in detail elsewhere (Bopp et al. 2009; Spoerri et al. 2010). Briefly, this cohort is based on two censuses carried out respectively in 1990 and 2000. As participation in censuses is mandatory, the Swiss Federal Office of Statistics assessed the coverage at 98.6%. We selected in the SNC all female participants aged over 18 and less than 65 and at work at the date of either of the two censuses.

### Follow-up

At the time of analysis (October 2019), the SNC data had been matched with the national mortality and migration files until Dec 31st 2014. Women aged over 18 and less than 65 on Dec 4th 1990 (the official 1990 census date) and at work at this date were followed up from Dec 4th 1990. Women aged over 18 and less than 65 on Dec 4th 2000 (the official 2000 census date) and at work at this date and who were not part of 1990 cohort were followed up from Dec 4th 2000. The end of follow-up date was defined as the minimum of following dates: the date of death, the date of loss to follow up (e.g., immigration), Dec 31st 2014.

### Data and data management

We abstracted a large number of variables from the SNC at each of the censuses. Some variables characterizing the subjects were independent of the censuses like date of birth, the education level in three categories (compulsory education or less, secondary education, tertiary education). Other socio-demographic variables could vary according to the censuses like the nationality (Swiss/non-Swiss), the religious affiliation, the geographical region of residence within Switzerland, the main language, the civil status, the working commune summarized as city centers, others, not available and finally the Swiss neighborhood economic index (Panczak et al. 2012). Note that the religious affiliation was regrouped into three groups according to its attitude towards suicide (strong opposition—Catholic/Jewish/Islamic-, tolerant—reformed Protestant-, no religious affiliation, other or unknown).

Finally the job characteristics consisted in the job type itself, the economic sector, the socio-economic status and the weekly hours worked. The job title was available as coded according to the 1988 version of the 4 digit the International Standard Classification of Occupation (ISCO code) of the International Labour Office. The economic sector of activity was coded according to the Swiss General Classification of Economic Activities (NOGA) published by the Swiss Federal Statistical Office, a classification close to the European Union Statistical classification of economic activities (NACE). The socio-economic status (SES) was defined in nine categories (1: top management, 2: other self-employed, 3: professionals, 4: skilled workers 5: unskilled workers, 6: in paid employment not otherwise classified, 7–9 categories not at work) while the weekly hours were recoded as < 35 h, 35–45 h, > 45 h.

Given the limited number of events in the present cohort, we recoded some variables. Thus, both the weekly working hours and the NOGA activity code were regrouped. In the same way, the job code available in 4 digits were considered in 2 digits (see Table 1) and when the number of events was lower than ten, it was regrouped with the nearest job code. However, in order not to lose any possibly relevant signal, we identified a short list of 3 or 4 digits job codes for which an excess in suicide SMR in comparison with the Swiss mortality rates had been described in the supplemental tables in the descriptive paper (Guseva Canu et al. 2019).

**Table 1:** Selected multiple negative binomial regression model for non-occupational confounders

		RR 95% Confidence interval
**Age (*p*** **< 0.0001)**		
18–34	Ref.	
35–44	1.88	(1.59–2.22)
45–54	2.00	(1.69–2.38)
55 +	1.93	(1.60–2.33)
**Period (*p*** **= 0.009)**		
1990–1999	Ref.	
2000–2014	0.77	(0.63–0.94)
**Age × period (*p*** **< 0.0001)**		
35–44/2000–2014	0.74	(0.57–0.95)
45–54/2000–2014	1.08	(0.84–1.38)
55 + /2000–2014	1.40	(1.08–1.81)
**Swiss regions (*p*** **= 0.004)**		
Lemanic region	Ref.	
Mittelland region	0.97	(0.85–1.11)
Northwest Switzerland	0.91	(0.78–1.05)
Zürich	1.01	(0.88–1.15)
Eastern Switzerland	0.98	(0.85–1.13)
Central Switzerland	0.91	(0.77–1.08)
Ticino	0.49	(0.36–0.66)
**Civil status (*p*** **< 0.0001)**		
Single	Ref.	
Married	0.54	(0.49–0.60)
Widowed	0.81	(0.63–1.06)
Divorced	0.97	(0.84–1.10)
**Highest training (*p* = 0.0363)**		
Compulsory education	Ref.	
Secondary education level	0.98	(0.88–1.09)
Tertiary education level	0.89	(0.78–1.03)
Not known	1.68	(1.07–2.63)
**Nationality (*p* = 0.0057)**		
Swiss	Ref.	
Non-Swiss	0.84	(0.75–0.95)
**Working commune type (*p* = 0.0001)**		
Not known	Ref.	
City centers	0.71	(0.51–0.97)
Other	0.61	(0.44–0.83)
**Intercept**	15.54	(10.94–22.09)

As the aim of this analysis was to assess the effect of occupational variables, we excluded all periods for which this information was missing. Thus, for instance, a subject entered the follow-up only in 2000 if her ISCO or NOGA code was missing in the 1990 census.

### Statistical methods

The statistical analysis relied on the cross-tabulation of the person-years and the number of deaths by suicide according to the selected independent variables. The data consisted therefore in the number of suicides and the corresponding person-years for each combination of independent variables.

The suicide incidence rate per 100,000 person-years (IR), that is the number of suicide occurring in any time cell defined by the independent variables, divided by the total person-years cumulated in this time-cell, was analyzed using the negative binomial regression. This method is an extension of the Poisson regression taking into account overdispersion, i.e., the fact that because of unmeasured factors, the variance of the rates may be larger than predicted by the usual Poisson approximation, which considers only the sampling variability. In practice, the models were selected using the faster Poisson regression and the final models were then fitted using negative binomial regression. The results for each factor were expressed as relative risks with respect to a reference category and as marginal predictions of the rates, which are model-predicted rates adjusted (marginalized) on all the other factors included in the model.

The analysis started with the joint modeling of the available non-occupational variables. This model was based on a stepwise inclusion of variables with a *p *value for inclusion lower than 0.20. This step led to the selection of a series of potentially confounding variables for the second step. The latter consisted in describing the different occupational variables each at the time after adjustment on the selected potential confounders followed by a stepwise selection of the selected job-related variables. Finally, the 3- or 4-digit job codes identified as in excess in the first descriptive study were included to check whether these excesses could be explained by the identified cofactors. The p values for model-comparisons were based on likelihood-ratio statistics referred to the chi-2 distribution.

## Results

Overall the cohort included 1,771,940 women and 24,959,598 person-years and 2526 deaths by suicide which corresponded to an overall crude suicide rate of 10.1 suicides per 100,000.

**Fig. 1:**
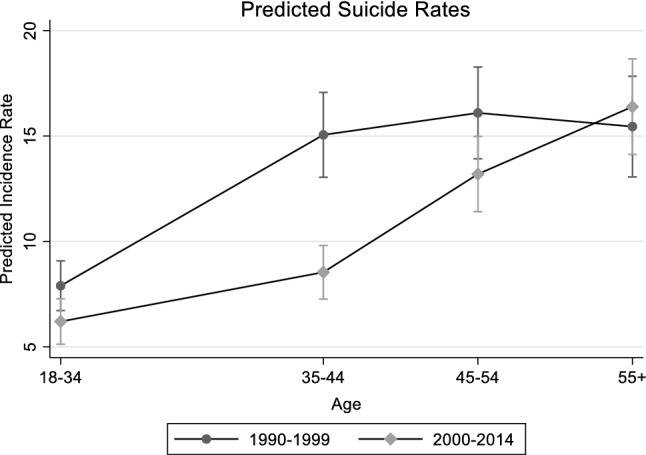
Model-predicted suicide rates and 95% Confidence Intervals according to age category and period of follow-up marginalized on Swiss regions, civil status, religion, highest training level, nationality and type of commune

The non-occupational variables selected in the first step of the analyses were the following (see Table 1 for the full final regression table): Age (rates increased with age), period (lower rates in recent periods but only among women aged less than 55 (see Fig. 1)—the age-period interaction was thus statistically significant—the Swiss regions, the civil status, the highest training level, the nationality, the religious affiliation and the type of working communes.

It must be noted that the rates decreased with the neighborhood economic index, though not significantly. This is consistent with lower rates among women working in city centers. Unfortunately, this index was only available from 2000 on.

**Fig. 2:**
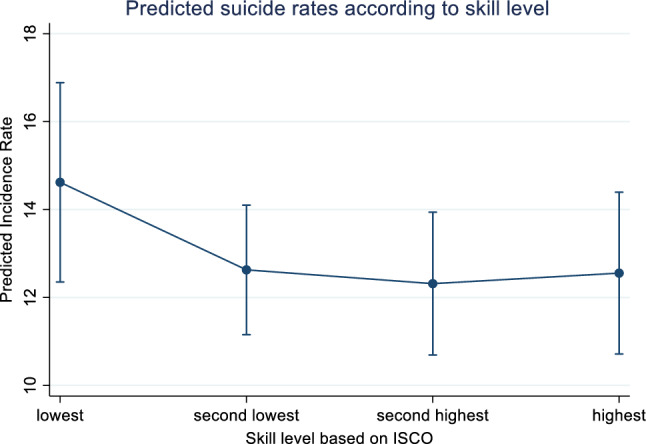
Model-predicted rates and 95% Confidence Intervals according to the skill level marginalized on Swiss regions, civil status, religion, highest training level, nationality and type of commune

With respect to job codes and classification, the first noteworthy result was that the adjusted suicide rates did not differ to a large extent according to skill level (*p* = 0.22, see Fig. 2) although the highest rates occurred in the lowest skill levels.

The results of the analyses with respect to the 2-digit ISCO codes, are displayed in Table 2. Overall, the difference in job codes is at the limit of statistical significance (*p* = 0.059) but some noteworthy features were apparent. The highest rates were observed in “Drivers and mobile plant operators” based on 13 cases. A noteworthy feature was that “Life science and health professionnals” have the highest (15.3) rates among the occupations with the highest skill level, “Life science and health associate professionnals” had the highest (12.0) rates among the occupations with the second highest skill level. Moreover the category “Personal and protective services including “Personal care and related workers” had one of the highest rates (13.0) in the second lowest skill level. The lowest rates were observed in “Managers of small enterprises”. Teaching professionnals and associate professionnals had also consistently low rates.

**Table 2:** Number of subjects, person-years and suicides, adjusted marginal rates and relative risks according to the ISCO-2digit job classification

	*n* subjects	Person-years	*n* suicides	Rates per 100,000 person-years (95%CI)		Adjusted RRs (95%CI)	
ISCO 2d (*p* = 0.059)							
Legislators, senior officials	25′110	93′363	10	12.63	(4.74–20.52)	1.16	(0.62–2.17)
Legislators and senior officials	13′076	46′951	7	15.07	(3.85–26.29)	1.38	(0.65–2.92)
Corporate managers	79′656	861′188	86	10.78	(8.39–13.18)	0.99	(0.78–1.24)
Managers of small enterprises	26′399	318′911	23	8.32	(4.86–11.79)	0.76	(0.50–1.16)
Science professionnals	17′461	220′472	24	12.72	(7.53–17.91)	1.16	(0.77–1.76)
Life science and health professionals	15′961	223′068	30	16.20	(10.13–22.27)	1.48	(1.01–2.18)
Teaching professionals	64′213	814′391	74	10.25	(7.78–12.73)	0.94	(0.73–1.21)
Other professionals	75′287	952′267	118	13.61	(10.93–16.30)	1.25	(1.01–1.54)
Physical and engineering science associate professionnals	44′728	549′673	52	10.57	(7.60–13.53)	0.97	(0.73–1.29)
Life science and health associate professionnals	158′069	2′164′658	217	11.97	(10.17–13.77)	1.10	(0.93–1.29)
Teaching associate professionals	82′213	1′122′764	99	10.35	(8.16–12.54)	0.95	(0.76–1.18)
Other associate professionals	164′151	2′016′287	212	11.70	(9.92–13.48)	1.07	(0.91–1.26)
Office clerks	387′129	5′019′195	484	10.92	(9.64–12.20)	Ref.	
Customer services clerks	69′820	844′399	99	13.43	(10.60–16.26)	1.23	(0.99–1.53)
Personal and protective services worker	247′195	3′023′053	325	13.07	(11.43–14.70)	1.20	(1.04–1.38)
Models, salespersons and demonstrators	182′988	2′205′123	208	11.18	(9.46–12.90)	1.02	(0.87–1.21)
Agricultural and fishery workers	52′841	635′907	48	9.99	(7.03–12.94)	0.91	(0.68–1.24)
Extraction and building trades workers	5′264	58′867	7	15.08	(3.86–26.30)	1.38	(0.65–2.91)
Metal, machinery and related trades workers	14′022	151′843	12	9.82	(4.22–15.43)	0.90	(0.51–1.60)
Precision, handicraft, craft printing a	20′723	252′537	29	13.05	(8.21–17.88)	1.19	(0.82–1.74)
Other craft and related trades workers	34′541	382′972	28	9.30	(5.80–12.79)	0.85	(0.58–1.25)
Machine operators and assemblers	27′677	295′424	31	13.13	(8.42–17.84)	1.20	(0.83–1.74)
Drivers and mobile plant operators	4′989	60′319	13	22.75	(10.28–35.22)	2.08	(1.20–3.62)
Sales and services elementary occupations	92′480	1′027′331	106	13.65	(10.85–16.45)	1.25	(1.00–1.56)
Labourers in mining, construction, and manufacturing	172′440	1′614′665	184	13.61	(11.36–15.85)	1.25	(1.04–1.49)

Adjusted on Age × Period, Swiss regions, civil status, religion, highest training level, nationality, type of commune

**Fig. 3:**
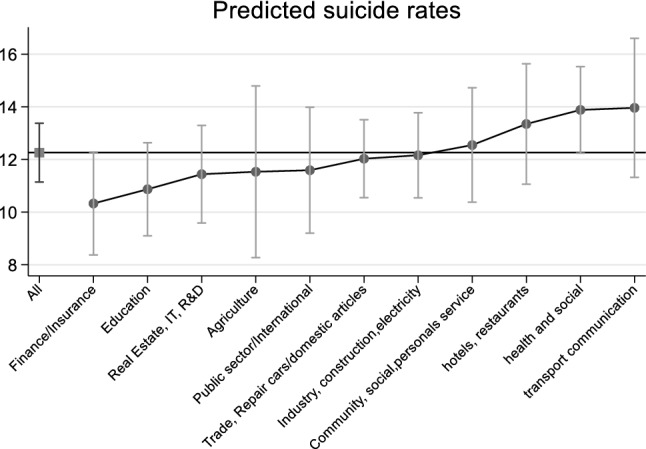
Model-predicted suicide rates and 95% Confidence Intervals according to sector of activity (regrouped NOGA code) marginalized on Age x Period, Swiss regions, civil status, religion, highest training level, nationality, type of commune

Overall, the differences according to the code for sector of activity were again at the limit of statistical significance (*p* = 0.049). Figure 3 shows the adjusted marginal rates according to the activity code. Table 3 displays the number of subjects, person-years and suicides, adjusted rates and relative risks. When compared to the reference category (trade, repair car/domestic articles) which contained the highest number of person-years, the single statistically significant category was the sector of Health and social activities (N) for which the adjusted rate exceeded 14.0/100’000.

**Table 3:** Number of subjects, person-years and suicides, adjusted marginal rates and relative risks according to the NOGA code of the activity sector

	*n* subjects	Person-years	*n* suicides	Rates per 100,000 person-years		Adjusted RRs	
NOGA regrouped (*p* = 0.049)							
Finance/insurance (J)	123′194	1′582′697	141	10.41	(8.27–12.56)	0.86	(0.71–1.05)
Education (M)	165′310	2′199′706	202	11.05	(9.05–13.05)	0.91	(0.77–1.09)
Agriculture(A) fisheries (B)	56′845	664′063	56	11.66	(8.23–15.08)	0.96	(0.72–1.28)
Real estate, IT and R&D Business (K)	174′965	2′142′857	202	11.69	(9.58 to 13.79)	0.97	(0.82–1.15)
Administration, defence, social security (L) International (Q)	84′611	1′057′267	110	11.81	(9.20–14.42)	0.98	(0.79–1.21)
Trade, repair car/domestic articles (G)	402′197	4′984′685	486	12.09	(10.34–13.83)	Ref.	
Extractive (C), Manufacturing industries (D), utilities (E), building (F)	314′001	3′672′431	361	12.36	(10.46–14.25)	1.02	(0.89–1.18)
Community, social, personal service activities (O)	128′607	1′514′298	161	12.69	(10.29–15.09)	1.05	(0.87–1.26)
Hotels and restaurants (H)	136′204	1′605′360	164	13.42	(10.92–15.92)	1.11	(0.93–1.33)
Health and social activities (N)	327′400	4′326′250	505	14.04	(12.06–16.02)	1.16	(1.02–1.33)
Transport and communication (I)	93′748	1′209′769	138	14.16	(11.26–17.06)	1.17	(0.96–1.42)

Surprisingly, no difference in suicide rates was observed with respect to socio-economic status (*p* = 0.509)—data not shown.

Finally, the most significant (*p* = 0.008) occupational factor was the difference in suicide rates according the weekly hours at work (Table 4). Although the main observed difference was between the part-time workers (< 35 h) and the workers on a standard schedule (35–45 weekly hours), the high suicide rate (13.47) in women working more than 45 h is noteworthy. This factor is still statistically significant (*p* = 0.01—data not shown) when further adjusted on the job code.

**Table 4:** Numbers of subjects, person-years and suicides, adjusted marginal rates and relative risks according to the weekly working hours

	*n* subjects	Person-years	*n* suicides	Rates per 100,000 person-years (95%CI)		Adjusted RRs (95%CI)	
Working hours (*p* = 0.008)							
No information	127′363	1′268′326	137	13.71	(11.05–16.38)	1.15	(0.95–1.38)
1–35 h	805′505	10′378′856	1081	14.05	(12.32–15.78)	1.18	(1.07–1.29)
36–45 h	929′517	11′770′701	1141	11.96	(10.55–13.37)	Ref.	
> 45 h	132′722	1′541′497	167	13.47	(11.00–15.93)	1.13	(0.95–1.33)

Adjusted on Age × Period, Swiss regions, civil status, religion, highest training level, nationality, type of commune

The stepwise modelling of the occupational variable confirmed the differences in working hours both when further adjusted on the 2-digit job code or the sector of activity. However, the joint inclusion of the job-code and the sector of activity—data not shown—was not relevant due to the strong association between the two factors.

Finally, after adjustment on the non-occupational factors, on the sector of activity and working hours, three 3-digit jobs were in significant excess compared to all other workers.

- ISCO 243 Archivists, librarians and related information professionals—adjusted rate 20.0 (95%CI 9.7–30.3) based on 15 suicides—no subcategory standing out.
- ISCO 513 Personal care and related workers—adjusted rate 14.5 (95%CI 11.6–17.4) based on over 124 suicides. Within this category, child-care workers [ISCO 5131 – 9 suicides, adjusted rate 17.1 (95%CI 5.8–28.4), institution-based personal care workers [ISCO 5132–65 suicides, adjusted rate 14.7 (95%CI 10.8–18.5)] and unspecified care workers [ISCO 5130-33 suicides, adjusted rates 15.0 (95%CI 9.7–20.4)] are in highest excess.
- ISCO 832 Motor vehicle drivers—adjusted rate 22.2 (95%CI 9.4–35.0) based on 12 suicides all of which are either tram or bus drivers or unspecified.

## Discussion

The present study showed the importance of some occupational factors on the suicide mortality among working women after adjustment of all the available non-occupational factors. With respect to the job codes and sector of activity, the most striking features are the high mortality in the health sector and the transport and communication sector. These findings are corroborated by consistently high suicide rates among health professionals and personal care workers. Within the latter group, the suicide rate in the subgroup of institution-based personal care workers was in significant excess. In the transport sector, the significantly high suicide rates among motor vehicle drivers are noticeable, although based on a small number of cases. Of particular interest are the increased rates among part-time workers and to a lesser extent among women working long hours, features that persisted after adjustment on the job code or the sector of activity.

This study confirmed some of the results of our previous descriptive study, showing that motor vehicle drivers; personal care and related workers as well as manufacturing labourers are the most at risk occupation with respect to the suicide in Swiss women (Guseva Canu et al. 2019). Moreover, health and social activity, and transport and communication appeared to be economic sectors at excess risk of suicide, even after adjustment for occupational and non-occupational factors. This means that these two economic sectors should be considered for further etiological research and preventive interventions. On the other hand, the excess risk among writers and creative performance artists was not confirmed when adjusting for the non-occupational factors.

The evidence that nurses and physicians have higher rates of suicide compared to the general population and other occupations is consistent in both sexes. (Davidson et al. 2020; Gold et al. 2013; Guseva Canu et al. 2019; Hawton et al. 2011, 2001; Hem et al. 2005; King et al. 1994; Kolves and De Leo 2013; Kuhn and Flanagan 2017; Schernhammer and Colditz 2004). However, conversely to the general population and other occupations, female nurses have higher rates of suicide than male nurses (Davidson et al. 2020; Gunnarsdottir and Rafnsson 1995; Hawton et al. 2011; Kolves and De Leo 2013). Many factors are associated with nurses’ suicide, but the presence of known work-related problems is one of the strongest (Davidson et al. 2020). Work stressors in nurses include strained relationships with colleagues and managers, conflict in the workplace, lateral violence, feeling alone in a new job, feeling insufficiently prepared for the role, production pressure, lack of control over one's professional life, and violence in the workplace (Accardi et al. 2020; Davidson et al. 2018; Gunnarsdottir and Rafnsson 1995; LeGal et al. 2019). Nurses and physicians face with health-care system challenges, including documentation requirements of electronic health records that result in clinicians spending less time with patients, maintenance of certification, loss of autonomy, unhealthy workplace cultures and heavy workloads (Fink-Miller and Nestler 2018; Kowalski et al. 2020). The presence of mental health problems has also been highlighted among clinicians (Schmid et al. 2020) with differences in their treatment between nurses and physicians (Davidson et al. 2020; Schernhammer and Colditz 2004). Nurses appear to receive mental health treatment more frequently than physicians or the general population (Davidson et al. 2020). Medical license applications include questions that reinforce the stigma of psychological stress and discourage physicians from seeking appropriate care (Kuhn and Flanagan 2017). In Switzerland, nurses are 43-year-old in average and 93% of them are women (Longchamp et al. 2018). In the study by Addor et al. (2016), the main work-related reported problems were burnout (72% unsatisfied), limited participation in decisions (62%), inflexible working hours (51%), insufficient salary (50%), workload (40%) and difficulty in reconciling work and family (41%) (Addor et al. 2016). As personal or home stressors, the US nurses reported financial and relationship problems. In anonymous testing, they also reported drug and alcohol use by family members or by themselves (Davidson et al. 2020). This underlines the fact that substance use and addiction-related disorders are undoubtedly under-identified among American nurses, and probably also among Swiss nurses (Schmid et al. 2020).

The evidence regarding the excess risk of suicide in female motor vehicle drivers and in transport and communication activity branch more generally is very limited. Only in Denmark have motor vehicle drivers been ranked among the 20 most at-risk occupations on the basis of the age- and sex-standardized ratio (Agerbo et al. 2007). In Queensland and UK, the excess of suicide in this occupation was reported exclusively for men (Andersen et al. 2010; UK-ONS 2017). This occupation includes drivers of motorcycles, cars, taxis and van and, excluding truck drivers, is part of the transport activity branch which is considered as male-dominated (Agerbo et al. 2007). Working in male-dominated occupations was associated with significantly higher risk of suicide compared to female-dominated occupations, but again, only for men (Milner and King 2019). With respect to these data, our finding is new. However, despite the low number of cases, there is some evidence that this excess is genuine. The excess of suicide in this occupation was observed in both sexes in Switzerland (Guseva Canu et al. 2019) and remained statistically significant after adjustment for potential confounders. Most females deceased by suicide in this occupation were bus drivers. The Swiss transport personnel trade union (SEV) consistently documented in 2010 and 2018 the presence of work-related physical and psychosocial stressors and mental health problems in Swiss bus drivers, and particularly in women (SEV 2019). Driving buses is considered as very stressful work (SEV 2019). SFSO further confirmed that 60% of workers in transport activity branch are exposed to at least three types of psychosocial risk factors at work, and that women were more often exposed to low decisional latitude, high emotional demand, discrimination and violence (SFSO 2019a). This occupation with atypical hours and mostly long hours, may pose difficulties for some women in reconciling work and family demands. Moreover, the lack of separate toilet and changing facilities alongside night work with terminals in relatively isolated and sometimes insecure areas can explain why few women embrace this job, although their number increased from 1 to 8% in Switzerland between 2010 and 2018 (SEV 2019). Therefore, this population deserves an urgent attention and targeted preventive interventions to improve their working conditions and mental health in order to reduce the suicide risk.

The increased risk of suicide observed among female drivers and mobile plant operators is also uncommon according to the literature. We do not have data supporting this finding, except that in women deceased by suicide in this occupation, the prevalence of substance-related and addictive disorders and of accidents, poisoning, and traumas was one of the highest compared to all other occupations in Switzerland (Schmid et al. 2020). This occupation also belongs to the male-dominated occupations and shares the same difficulties as those encountered by female working in the transport activity branch. Similarly, female labourers in mining, construction, and manufacturing are exposed to difficult working conditions, combining multiple physical and psychosocial risk factors (Karasek 1979; Milner et al. 2018).

The increased suicide among part-time workers is the most noticeable observation of our study. In Switzerland, elementary occupations have the highest proportion (> 60%) of part-time workers, three fourth of whom are women (SFSO 2019b). However, this fact cannot explain the increased risk among part-time workers as the latter persists after adjustment on the job code. Part-time work causes social, professional, and economic inequalities in both sexes (Krone-Germann and Aymone de Chambrier 2011), but particularly in women, given persistent gender inequalities penalizing women in terms of pay and access to management positions. Because part-time work is often characterized by fixed-term contracts, irregular working hours, on-call work and poor social security benefits (Krone-Germann 2011) it can be considered as precarious employment. While more and more men (about 18%) choose working part-time for education or leisure purpose, for Swiss women, part-time work is often socially imposed (Krone-Germann and Aymone de Chambrier 2011). This is due to the particularity of the Swiss socio-cultural heritage, which remains strongly conservative. Motherhood is the most important determinant of part-time work among women, with an odds ratio (OR) of 14.56 when the child is < 3 years old, 9.93 with a child aged 4–12, and 1.95 for a child aged 18 and over, compared to women without children (SFSO 2019b). Age and education are also part-time determinants of part-time employment. According to the Swiss Federal Statistical Office (SFSO 2019b), part-time employment is more frequent among women aged 25–39 (OR = 1.93) and aged 55–64 (OR = 3.62) compared with women aged 15–24 years. Similarly, when considering women with tertiary education as a baseline, women having secondary I (OR = 1.09) and secondary II (1.15) are more likely to be part-time workers (SFSO 2019b). Due to a long liberal tradition non-intervention by the State in the private sphere, such as childcare and education, there is a lack of childcare facilities and their costs are high compared to other countries. This leads Swiss mothers, especially those living in low-income households, to give up or reduce their paid work to care for their children (Giudici and Schumacher 2017)). In addition, the gender inequality between 16-week maternity leave (officially instituted in 2005!) and the almost non-existent paternity leave, encourages an unequal division of labour from the first weeks of family life. Finally, the progressive taxation of family income, discourages the economic participation of mothers, because from a purely financial point of view, it may be more advantageous for a family to reduce weekly hours worked (or even stop working) by one of the partners. Most often than not, it is the woman who reduces her working hours, because her income is often lower than that of her partner. This is particularly true when women are married and their partner has Swiss citizenship and tertiary education (Giudici and Schumacher 2017). Interestingly, among 40–54 year old women, 13.5% wish to work longer hours, which reflects the difference in under-employment among women and men (3.8%) (SFSO 2019b). Though according to the social surveys, Swiss women accept working part-time reconcile work and family (Krone-Germann 2011), socio-economic and structural factors seem to be the most important determinants of this “choice”.

Finally modelling the confounders mostly confirmed the effect of known risk factors like age, education, civil status and training. It is interesting to note the differential effect of religion which allowed us to distinguish suicide rates not only between religious affiliates and non-affiliates which has been demonstrated repeatedly, e.g., (Stack and Laubepin 2019) but also according to the attitude of these religions towards suicide with lower rates among affiliates of religions strongly condemning suicide. This suggests that the latter are either more effective in coping with distress or succeed in establishing life-saving beliefs (e.g., hell as punishment after suicide) among their followers (Ploderl et al. 2020).

This study has several strengths. First, the cohort contains the complete working female population of a whole, although admittedly small, country with an almost 100% coverage. Thus, on one hand, the number of suicides is large enough to detect even moderate effect sizes of occupational factors and on the other hand, there is no selection bias, which is rare in such a large cohort. A second major strength is the number of information items providing a complete picture of the social and to some extent personal environment of the study subjects. Some of these items (e.g., religion) are very rarely available in such large cohorts. A third strength is that all information items were collected prior to the event of interest. Thus, there can no recollection bias and moreover the information was provided by the subjects themselves.

The study has also some weaknesses. As mentioned in the introduction, there are some female-specific risk factors which may be dominant as causes of suicide. These include abortion, miscarriage or infant death at birth. In addition, there is a range of psychosocial factors that women are more likely to be exposed to, such as sexual violence, domestic violence and early puberty. None of these factors were of course available. A second weakness is that despite the size of the population, the number of deaths by suicides for any precise jobcode is quite limited. Thus, the observed excesses must be interpreted with the contextual information in mind. Finally, as in any mortality study based on deaths certificates, it is highly likely that a number of deaths by suicide have not been coded as such, thus limiting the statistical power. This underreporting is a quite general phenomenon (Li and Yip 2020) (Redaniel et al. 2011) although the Swiss National Programme on suicide prevention improved the reporting of suicides (Ostertag et al. 2019).

## Conclusion

Suicide among working women depended on work-related factors even taking into account other socio-demographic factors. Women with part-time work and in the care sector should be targeted for any work-related prevention.

## Data Availability

This work was conducted in the framework of the Swiss National Cohort nested study contract no. 2365. This contract stipulates that after the final publication, the data will no longer be available even to the authors. Access to the data can therefore only be with the Swiss National Cohort.
